# Functional Role of Dietary Intervention to Improve the Outcome of COVID-19: A Hypothesis of Work

**DOI:** 10.3390/ijms21093104

**Published:** 2020-04-28

**Authors:** Giovanni Messina, Rita Polito, Vincenzo Monda, Luigi Cipolloni, Nunzio Di Nunno, Giulio Di Mizio, Paolo Murabito, Marco Carotenuto, Antonietta Messina, Daniela Pisanelli, Anna Valenzano, Giuseppe Cibelli, Alessia Scarinci, Marcellino Monda, Francesco Sessa

**Affiliations:** 1 Department of Clinical and Experimental Medicine, University of Foggia, 71122 Foggia, Italy; 2 Department of Experimental Medicine, Section of Human Physiology and Unit of Dietetics and Sports Medicine, Università degli Studi della Campania “Luigi Vanvitelli”, 80138 Naples, Italy; 3 Department of History, Society and Studies on Humanity, University of Salento, 73100 Lecce, Italy; 4 Department of Law, Forensic Medicine, Magna Graecia University of Catanzaro, 88100 Catanzaro, Italy; 5 Department of General Surgery and Medical-Surgical Specialties, University of Catania, 95121 Catania, Italy; 6 Department of Mental Health, Physical and Preventive Medicine, Clinic of Child and Adolescent Neuropsychiatry, Università degli Studi della Campania “Luigi Vanvitelli”, 81100 Caserta, Italy; 7 Department of Education Sciences, Psychology and Communication, University of Bari, 70121 Bari, Italy

**Keywords:** COVID-19, IL-6, adiponectin, ω-3 PUFAs, lung infections, diet therapies

## Abstract

Background: On the 31 December 2019, the World Health Organization (WHO) was informed of a cluster of cases of pneumonia of unknown origin detected in Wuhan City, Hubei Province, China. The infection spread first in China and then in the rest of the world, and on the 11th of March, the WHO declared that COVID-19 was a pandemic. Taking into consideration the mortality rate of COVID-19, about 5–7%, and the percentage of positive patients admitted to intensive care units being 9–11%, it should be mandatory to consider and take all necessary measures to contain the COVID-19 infection. Moreover, given the recent evidence in different hospitals suggesting IL-6 and TNF-α inhibitor drugs as a possible therapy for COVID-19, we aimed to highlight that a dietary intervention could be useful to prevent the infection and/or to ameliorate the outcomes during therapy. Considering that the COVID-19 infection can generate a mild or highly acute respiratory syndrome with a consequent release of pro-inflammatory cytokines, including IL-6 and TNF-α, a dietary regimen modification in order to improve the levels of adiponectin could be very useful both to prevent the infection and to take care of patients, improving their outcomes.

## 1. Background

On the 31 December 2019, the World Health Organization (WHO) was informed of a cluster of cases of pneumonia of unknown origin detected in Wuhan City, Hubei Province, China. About one month later (on 8 January 2020), the Chinese authorities declared the identification of a new type of coronavirus, informing the WHO a few days later that the outbreak was associated with exposure in a seafood market in Wuhan City. The infection spread firstly in China and then in the rest of the world, and on the 11th of March, the WHO declared that COVID-19 was a pandemic.

Coronaviruses (CoVs) belong to the subfamily Orthocoronavirinae in the family of Coronaviridae in the order Nidovirales, and this subfamily includes α-coronavirus, β-coronavirus, γ-coronavirus, and delta-coronavirus [1]. Coronaviruses primarily cause enzootic infections in birds and mammals and, in the last few decades, have shown to be capable of infecting humans as well [2]. In human infections with highly virulent respiratory viruses—such as avian influenza H5N1, H7N9, Severe Acute Respiratory Syndrome (SARS) coronavirus, and Coronavirus Disease-19 (COVID-19)—immunopathogenesis caused by the overproduction of pro-inflammatory cytokines may play an essential role in disease progression and mortality [3]. Several recent studies have reported that COVID-19 caused the destruction of the pulmonary parenchyma, including interstitial inflammation and extensive consolidation, similarly to the previously reported coronavirus infection [4,5]. During coronavirus infection, it was observed that the lungs increased in weight, with a mild pleural effusion of clear serous fluid, named pulmonary edema, and extensive consolidation [6,7]. In some areas, there was interstitial thickening, with mild-to-moderate fibrosis, but a disproportionately sparse infiltrate of inflammatory cells (mainly histiocytes, including multinucleated forms, and lymphocytes) [8]. A dilatation of the airspaces was observed, as was focal honeycombing fibrosis. An intra-alveolar organization of exudates was described, and the formation of granulation tissues in the small airways and airspaces was reported. These lesions were typically located in the sub-pleural region, and the cellular component mainly consisted of histiocytes, as reported in a previous paper [9]. Xu et al. described in their case report the pathological findings of COVID-19 associated with acute respiratory distress syndrome. At the X-ray investigation, they detected a rapid progression of bilateral pneumonia. The biopsy samples were taken from the lung; the histological examination showed bilateral diffuse alveolar damage with cellular fibromyxoid exudates [6].

Considering that the mortality rate of COVID-19, about 5–7% [10], and the percentage of positive patients admitted to intensive care units being 9–11% [11], it should be mandatory to consider and take all necessary measures intended to contain the viral infection.

A recent study analyzed the data of 150 COVID-19 patients, with the aim of defining the clinical predictors of mortality. The results obtained from this study suggest that COVID-19 mortality might be due to virus-activated “cytokine storm syndrome”, considering that the plasma levels of IL-6 were higher in deceased patients compared to in discharged subjects [12].

**Figure 1:**
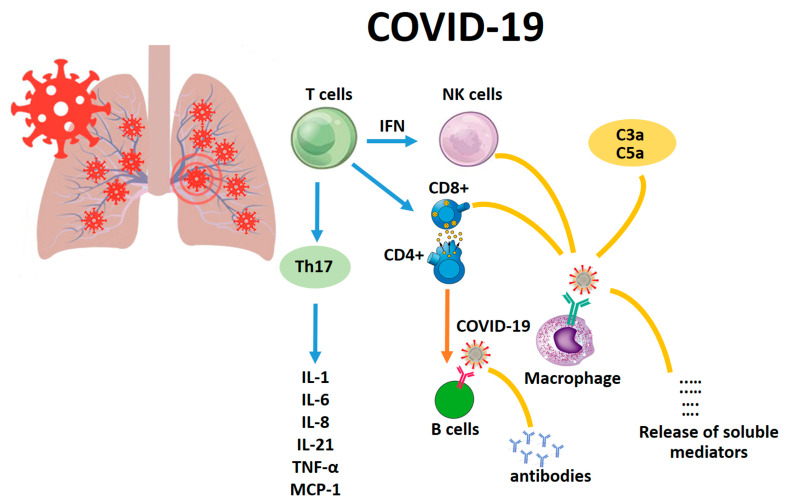
The main immunological response to COVID-19.

Considering that a detailed study has not been performed on the immunological response to COVID-19, the only way to discuss this thematic is to refer to previous knowledge about SARS-CoV and MERS-CoV. The first response is obtained through pattern recognition receptors (PRRs) including C-type lectin-like receptors, Toll-like receptors (TLR), NOD-like receptors (NLR), and RIG-I-like receptors (RLR). Moreover, several inflammatory factors are expressed such as IL-6 and TNF-α; moreover, the synthesis of type I interferons (IFNs) is activated, and these exert their actions against virus diffusion, accelerating macrophage phagocytosis [13] (Figure 1).

In the light of these considerations and the recent evidence in different hospitals suggesting IL-6 and TNF-α inhibitor drugs as a possible therapy for COVID-19, this review aims to highlight how a dietary intervention could be useful to prevent the infection and/or to ameliorate the outcome during therapy.

## 2. The Pivotal Role of IL-6 and TNF-α in Lung Infections

The first laboratory report about COVID-19 patients indicated several parameters that were found to be altered in blood samples; for example, D-dimer, neutrophil count, blood urea, and creatinine levels were significantly higher. In the same way, several cytokines such as IL-6 and TNF-α were overexpressed, indicating the immune status of the patients [14].

IL-6 represents pro-inflammatory signaling produced by adipose tissue; for this reason, this endocrine cytokine could be important in regulating the host response during acute infection [15].

Several papers have described the essential role of IL-6 in generating a proper immune response during different kinds of viral infection in the pulmonary tract. Others link this cytokine to an exacerbation of viral disease. These latter findings support the hypothesis that IL-6 upregulation during viral infections may promote virus survival and the exacerbation of the clinical disease [16,17]. Indeed, IL-6 has a pleiotropic function, and it is produced in response to tissue damage and infection. In particular, at the pulmonary level, innate and adaptative immune cell proliferation is strongly influenced by this cytokine. After targeting its specific receptor, IL-6 starts a cascade of signaling events mainly associated with the JAK/STAT3 activation pathway, promoting the transcription of multiple downstream genes related to cellular signaling processes, including cytokines, receptors, adaptor proteins, and protein kinase [15]. Furthermore, it has been reported that IL-6 is an essential factor for the survival of mice with a viral infection. This cytokine promotes the optimal regulation of the T-cell response, inflammatory resolution, tissue remodeling promoting lung repair, cell migration, and the phagocytic activities of macrophages, as well as preventing virus-induced apoptosis in lung epithelial cells. However, experimental scientific evidence also suggests potential adverse consequences that increased levels of IL-6 might have on the cellular immune response against viruses. In this context, different possible mechanisms involving this cytokine might affect viral clearance, ultimately favoring the establishment of a persistent viral state in infected hosts [18,19].

Tumor necrosis factor is a cell-signaling protein (cytokine) involved in systemic inflammation, released predominately from macrophages, but it is also released from a variety of other immune cells. It has been well described that during infection with the influenza virus, the expression of TNF-α in lung epithelial cells was higher, exerting powerful anti-influenza virus activity [20]. In an animal model, it has been demonstrated that TNF-α plays a pivotal role in the development of pulmonary fibrosis. TNF-α signals via two receptors, TNF-RI and TNF-RII; the first receptor (TNF-RI) promotes intracellular signaling involving c-Jun N-terminal kinase (JNK) and nuclear factor (NF)-κB, while the other receptor, TNF-RII, promotes TNF-RI–dependent cell death, without directly inducing apoptosis. Although both receptors are broadly expressed, it is known that the majority of inflammatory signaling is elicited through TNF-RI [21]. In an in vitro model, it has been described that serine/threonine kinases can phosphorylate TNF-RI and its molecules, preventing tyrosine phosphorylation [22,23,24].

In patients with COVID-19, the high serum levels of IL-6 and TNF-α are negatively correlated to T cells; contrariwise, it has been demonstrated that T cell levels were restored by reducing IL-6 and TNF-α concentrations [25]. These findings suggested that these cytokines could represent important targets of anti-COVID-19 therapies.

## 3. Adiponectin Function in Lung Infections

Through the secretion of adipokines, adipose tissue participates in the regulation of several pathophysiological processes in many organs and tissues. Among the adipokines, adiponectin is the most relevant. Adiponectin is one of the most abundant circulating adipocytokines, accounting for 0.01% of total serum protein. Adiponectin is an important regulator of cytokine responses, and this effect is isoform-specific. It is involved in a wide variety of physiological processes, including energy metabolism, inflammation, and vascular physiology. These effects are mediated by two atypical, widely expressed seven-transmembrane receptors, AdipoR1 and AdipoR2 [26]. Adiponectin has beneficial effects in cardiovascular systems and blood vessels, protecting these tissues through the inhibition of pro-inflammatory and hypertrophic responses and stimulation of endothelial cell responses [27]. Adiponectin circulates as three different isoforms (low molecular weight—LMW, medium molecular weight—MMW, and high molecular weight—HMW) [28].

Infectious diseases are characterized by an increased production of adiponectin. Several papers suggest that adiponectin may be related to disease activity and/or severity in different conditions such as rheumatoid arthritis, osteoarthritis, and systemic lupus erythematosus. Since adiponectin has been found to display both pro- and anti-inflammatory activities, controversial findings have been observed regarding the role of total adiponectin in systemic autoimmune and inflammatory joint diseases. For this reason, the relative contribution of each adiponectin isoform to the inflammatory response and joint and/or tissue damage requires further study [29]. It is reported that adiponectin is regulated by transcription factors in adipose tissue, such as peroxisome proliferator-activated receptor-γ (PPAR-γ) [30]. During viral infections, it has been reported that the role of the predisposition of hosts is also important, as well as their state of health and nutrition. Indeed, it is well known that white adipose tissue is considered an endocrine source of biologically active substances with local and/or systemic action, called adipokines. The inappropriate secretion of adipokines seems to participate in the pathogenesis of obesity-related diseases, including endothelial dysfunction, inflammation, and atherosclerosis [31,32,33].

The biological function of adipokines in lung diseases seems to be mainly related to the inflammatory process. In particular, the intercorrelation between adipose tissue and the lung has become evident as the involvement of adiponectin has been demonstrated in several lung diseases such as Chronic Obstructive Pulmonary Disease (COPD), emphysema, and cancer [34]. In fact, with specific regard to COPD, a low-grade inflammatory state has been demonstrated [35,36,37]. Moreover, increasing evidence suggests that adiponectin also exerts a crucial role in the vascular endothelium, maintaining vascular homeostasis and protecting against vascular dysfunctions. Altogether, these findings support the anti-inflammatory role of adiponectin in COPD and, in general, in other lung diseases [38].

The critical role of adiponectin in the pathophysiological conditions of the lung is also supported by the modulation of AdipoRs with the downregulation of AdipoR2. It has been described that the adiponectin oligomerization state is altered in COPD; moreover, the presence of AdipoR1 and AdipoR2, with a lower expression of AdipoR2 compared to AdipoR1, in lung tissue [39] has been demonstrated. The low expression of AdipoR2 could suggest a specific role of this receptor, mainly implicated in adiponectin’s effects on inflammation and oxidative stress. Mainly, it has been observed that higher levels of adiponectin are associated with a significant and specific increase in HMW adiponectin, representing the most biologically active forms. Thus, HMW adiponectin increases IL-6 secretion in human monocytes and human monocytic leukemia cell lines but does not suppress lipopolysaccharide (LPS)-induced IL-6 secretion. Byn contrast, LMW adiponectin reduces LPS-mediated IL-6 release and also stimulates IL-10 secretion [40]. Furthermore, several in vitro studies have demonstrated that adiponectin in the A549 adenocarcinoma human alveolar basal epithelial cell line has an essential apoptotic effect and also reduces the production of pro-inflammatory cytokines such as TNF-α, blocking NF-κB nuclear translocation [41,42].

Indeed, adiponectin can reduce innate and adaptive immune cell proliferation and polarization, also blocking the production of pro-inflammatory cytokines such as TNF-α, IL-2, and IL-6, and enhancing that of anti-inflammatory cytokines such as IL-10, with a decrease in the phosphorylation of AMPK, p38, ERK1/2, and c-JNK [43,44,45,46]. Data from in vitro studies on lung cells were consistent with an anti-inflammatory function of adiponectin, and adiponectin-deficient mouse models developed lung function impairments and systemic inflammation [47]. The possible role of adiponectin in inflammatory pulmonary diseases, such as asthma and chronic obstructive pulmonary disease (COPD), and in critical illnesses has been the subject of recent investigations. Particularly, the HMW isoform has a specific role in pulmonary diseases and critical illnesses, even if its role should be better clarified [48,49].

An interesting study reported that systemic adiponectin concentrations in humans fall during the acute phase of lung infection: particularly, during the early phase, the pro-inflammatory state is generated by the high systemic TNF-α and IL-6 concentrations, with the subsequent inhibition of adiponectin production. Contrariwise, it has been described that the reduction in TNF-α and IL-6 factors generates a corresponding bounce-back in systemic adiponectin concentrations [50]. Although it is still unclear whether the modulation of systemic adiponectin or its signaling pathways has any therapeutic benefit in pulmonary or critical illnesses, it may serve as a novel therapeutic or preventative tool for these illnesses in the future. One obvious pharmaceutical treatment would be the exogenous administration of adiponectin by the inhalational or intravenous route. Although this has been tried in mouse models [51], the problems to be overcome prior to human administration include establishing what the biologically active molecule is and what role post-translational modifications have upon its function, and the associated difficulties in generating biologically active molecules on a large scale.

Considering the difficulty linked to the direct administration of adiponectin, in the last few years, other drugs have been used that indirectly improve adiponectin production. For example, a synthetic ligand of peroxisome proliferator-activated receptors can increase adiponectin mRNA in adipocytes, improving the production and secretion of adiponectin [52,53,54,55]. Moreover, other drugs such as fibrates can increase systemic adiponectin levels by enhancing PPAR-γ activity [56,57]. Another way to improve adiponectin levels is the use of angiotensin converting enzyme inhibitors [58,59,60]. Furthermore, it is possible to stimulate adipocyte differentiation [61] and the activation of PPAR [62]. Finally, it has been described that calcium channel blockers [63] and a central-acting anti-hypertensive agent [64] also increase systemic adiponectin concentrations [65].

The possibility to improve the action of adiponectin through diet is intriguing; it has been described that nutritional interventions may help to regulate systemic adiponectin concentrations. In an animal model, it has been demonstrated that a diet with a high concentration of polyunsaturated fatty acids and supplemented with ω-3 can improve the plasma levels of adiponectin, increasing gene expression [66]. On the other hand, in humans, adiponectin levels are positively associated with a healthy lifestyle and the Mediterranean diet, even if the mechanisms of action are not completely known [66]. Finally, in light of these considerations, in COVID-19 therapy, it could be very useful to combine drug therapy with a specific diet regimen.

## 4. ω-3 PUFAs and Lung Infections

Another important mediator involved in the immune response and influenced by nutrition are fatty acids, in particular, ω-3 PUFAs [67,68]. In fact, during bacterial and viral infections, they are able to act on immune cells and regulate diverse inflammatory processes. ω-3 PUFAs are known to have anti-inflammatory properties and play an essential role in the resolution of inflammation [69].

In several lung infections, the administration of PUFA can ameliorate the outcome of the patient in acute pneumonia. Sharma et al. reported in their study that the dietary supplementation of ω-3 PUFA can exert an overall beneficial effect against acute pneumonia through the upregulation of the host’s specific and nonspecific immune defenses [70]. ω-3 polyunsaturated fatty acids (PUFA, ω-3-fatty acids), the key components of fish and flaxseed oils, are increasingly consumed by the public because of their potential health benefits and can be used clinically for the treatment of metabolic, cardiac, inflammatory, and autoimmune diseases [71]. However, numerous studies have shown that these compounds are immunoregulatory and immunosuppressive and thus may increase susceptibility to infection. While reports suggest that ω-3 PUFAs may have beneficial effects against extracellular pathogens, few studies have been performed on systemic viral infections in mammals. Jones and Roper described in their study that a diet rich in ω-3 PUFAs did not significantly lower survival of the vaccinia virus infection, at least with short-term (~6 week) feeding in mice [71].

ω-3 PUFAs are metabolized into various mediators possessing anti-inflammatory properties such as resolvins and protectins. It is known that ω-3 PUFAs can reduce NF-κB activation by preventing nuclear p65 NF-κB translocation. Furthermore, ω-3 PUFAs minimize the activation of ERK1/2 MAPK, also reducing COX-2 production. The ω-3 PUFA-derived lipid mediator could markedly attenuate influenza virus replication via the RNA export machinery. In addition, the treatment of protectin D1 with peramivir could completely stop mouse mortality [72].

ω-3 supplementation was previously studied in Acute Respiratory Distress Syndrome (ARDS). Singer and Shapiro suggested that the enteral administration of natural antioxidant substances could improve oxygenation and clinical outcomes in ICU patients [73]. A systematic review performed in 2015 reported a positive effect only for patients suffering from ARDS with high mortality [74]. A more recent meta-analysis highlighted the importance of clinical trials in order to clarify the use of ω-3 fatty acids and antioxidants in patients with ARDS to ascertain the positive effects in order to reduce the lengths of ICU stays and the numbers of days spent on ventilators [75].

Although the role of ω-3 supplementation in ARDS should be better clarified, its pivotal role in reducing reactive oxygen species and pro-inflammatory cytokines, such as TNF-α, IL-1β, IL-6, and IL-8 [76], is well known.

Therefore, ω-3 PUFAs, including protectin D1, which is a novel antiviral drug, could be considered for potential interventions for COVID-19.

## 5. Other Dietary Constituents and Lung Infections

As previously described, other dietary constituents can be used to improve the patients’ outcomes during lung infection, regulating the inflammatory response. Among these, antioxidants play an important role in protecting lung cells against viruses and bacteria. Viral infection leads to an increase in the intrapulmonary oxidative burden. In many diseases, the balance between oxidants and antioxidants (redox balance) is altered, with severe consequences [77]. The pathophysiological mechanisms by which free radicals generate various types of stress—such as oxidative, nitrative, carbonyl, inflammatory, and endoplasmic reticulum stress—lead to lung inflammation and an altered lung immune response. In this scenario, dietary antioxidants may play an important role against lung oxidative stress [77]. Several studies reported the protective role of the antioxidants in lung infection and in lung inflammation [78,79].

In particular, vitamin C, polyphenols, and flavonoids can play a protective role in lung infections, being immune modulators and inflammatory mediators. Indeed, as reported by Carr et al., during infection, vitamin C levels may become depleted; for this reason, vitamin C supplementation can attenuate infection. Based on this evidence, these authors suggested a clinical trial with vitamin C infusion for the treatment of severe COVID-19 patients [80].

Among polyphenols, epigallo-catechin 3 gallate (EGCG) is the most potent ingredient in green tea and exhibits antibacterial, antiviral, antioxidative, anticancer, and chemo-preventive activities. Recently, numerous studies have investigated the protective effects of EGCG against asthma and other lung diseases such as COPD and lung pneumonia. EGCG may suppress inflammation and inflammatory cell infiltration into the lungs of asthmatic mice, and may also inhibit epithelial-mesenchymal transition EMT via the PI3K/Akt signaling pathway through upregulating the expression of phosphatase and tensin homolog (PTEN), both in vivo and in vitro [81].

Moreover, flavonoids can be used to attenuate lung injury in mice; it has been reported that they inhibit influenza virus and Toll-like receptor signaling, blocking NF-κB translocation [82].

Therefore, as summarized in Table 1, supplementation with vitamin C, flavonoids, and polyphenols can be tested in COVID-19 patients, both in order to prevent viral infection and to improve patients’ outcomes.

## 6. Discussion and Conclusions

During pulmonary infections, and particularly in COVID-19 patients, intracellular signaling leads to the production of pro-inflammatory cytokines, such as TNF-α and IL-6, which act in concert with chemoattractants, such as CXCL1 and CXCL2, to recruit polymorphonuclear leukocytes (PMNs) to the lungs, killing pathogens but generating fibrosis [83].

Another important consideration during COVID-19 infection is related to the modification of the secretory products of the upper and lower airways, which usually include mucin and pulmonary surfactant. During infection, mucin production is upregulated, with the function of preventing microbes from binding to and infecting epithelial cells [84]. The primary source of phospholipids (PLs) in the lung is pulmonary surfactant, synthesized and released by alveolar epithelial type II cells. The surfactant contains approximately 80–90% PLs, with fatty acid chains that can be oxidized during different challenges in the lung [85]. The oxidation of these PLs in the lung can occur in the setting of an increased oxidative stress situation, such as infection and inflammation [86]. The immune effects of oxidized phospholipids oxPLs during infectious diseases are inevitably dictated by the balance among activation, degradation, and scavenging. It has been shown that oxPLs are generated in the lung during several pulmonary infections, including influenza and avian influenza (H5N1), as well as SARS coronavirus, even if the mechanisms of action are not well known [87,88,89]. As reported by Imai et al., oxPL-induced inflammation is mediated by TLR4 and TRIF, driving an increase in IL-6 production [89]. It is intriguing to consider that oxPL-dependent defects in phagocytosis and ROS generation may lead to an increased susceptibility to respiratory infections [90]. Cholesterol is the major neutral lipid in pulmonary surfactant, in which it is thought to promote the spreading, mobility, and adsorption of surfactant films [91]. As previously documented, modulating adiponectin levels can be considered an important way to reduce cytokines levels; in this way, the adverse effects related to the COVID-19 infection should be attenuated. It is well described in animal models that the consumption of hyperlipidemic diets, rich in saturated fat, reduces the levels of adiponectin, while diets rich in polyunsaturated fatty acids and supplemented with ω-3 PUFA increase adiponectin levels, reducing pro-inflammatory cytokines [66].

Innate and adaptive immune responses are influenced not only by oxPLs and cholesterol but also by the fatty acid profiles of tissues in response to pharmacological agents and diet [92]. Several studies performed in animal models demonstrated how ω-3 PUFA uptake into the lung tissue influences outcomes associated with infection, promoting the resolution of inflammation [93]. In another study, ω-3 PUFAs reduced the levels of PMNs and lowered IL-6 levels in lung infections [94]. These positive effects remain controversial; for example, Jones and Roper reported that in their experimental model, no statistically significant differences were found among the diet regimens, with and without ω-3 PUFAs, with respect to the susceptibility of mice to viral infection, morbidity, viral organ titers, recovery time, or mortality [71].

In conclusion, it is well known that general treatments are very important to enhance the host immune response against RNA viral infection. In addition, the immune response has often been shown to be weakened by inadequate nutrition in many model systems as well as in human studies. However, the nutritional status of the host, until recently, has not been considered as a contributing factor to the emergence of viral infectious diseases. The recent reports about the pathogenesis of COVID-19 suggested that one of the most important consequences of this infection is the cytokine storm syndrome [95], which could be strictly linked with coagulopathy, generating acute pulmonary embolism caused by in-situ thrombosis [96,97]. Therefore, a great number of clinical trials are ongoing to define a useful therapy to attenuate cytokine storms [98].

**Figure 2:**
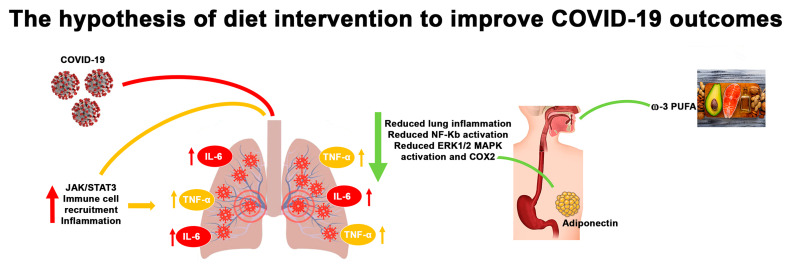
Adiponectin and ω-3 PUFAs reduce the lung inflammation that occurs following coronavirus infection, reducing IL-6 production, ERK1/2, and COX-2 activation and the nuclear translocation of NF-κB.

For these reasons, an adequate ω-3 PUFA intake may be a valid strategy against viral infection. Indeed, following the recommended intake of ω-3 PUFA, in the range of 0.5% and 2% of total calories (250 mg/day), may be important to protect against an excessive inflammatory response, also reducing IL-6 levels. This theory found important support in a recent study that demonstrated that ω-3 PUFA-derived lipid mediator protectins can suppress influenza virus replication through a mechanism that blocks the export of viral mRNA. Moreover, Imai demonstrated that this mediator can be used in combination with the antiviral peramivir, even at late time points in infection [99]. Nevertheless, the efficacy of ω-3 PUFAs at the clinical level is under investigation; for example, Hecker et al. described a beneficial effect for a diet regimen with ω-3 PUFAs, describing that the pro-inflammatory cytokine levels decreased after this diet regimen [100]. The suggested positive role in the outcome and prevention of the COVID-19 infection is summarized in Figure 2.

In addition, adiponectin plays a role in lung diseases and obesity; in the development and progression of lung disease and cancer, a pathogenic role of adiponectin was defined by both in vivo and in vitro studies. Recently, immunometabolic pathomechanisms have been identified as important factors determining and modulating lung function and disease. Particularly, adiponectin levels have been found to be greater in patients with COPD compared with in control patients, and adiponectin-deficient mice are protected from several lung diseases [101]. Moreover, it has been reported that adherence to the Mediterranean diet was associated with an increase in adiponectin levels, improving cardiovascular system functionality [102], particularly in elderly people [103]. These findings are only apparently contradictory to the first data about the mortality rate from COVID-19 infections in the Mediterranean area (such as in Italy and Spain) [104]. First of all, the data have been referred only to the tested population; moreover, it is well described that the presence of several comorbidities such as hypertension, diabetes, and cardiovascular diseases severely influenced the mortality rate reported in this area [105]. All these comorbidities can be counteracted with a correct dietary regimen. Therefore, both adiponectin and ω-3 PUFAs appear to be attractive biomarkers for monitoring lung disease progression.

Finally, considering that the COVID-19 infection can generate a mild or highly acute respiratory syndrome with a consequent release of pro-inflammatory cytokines, including IL-6 and TNF-α, a modification of the dietary regimen in order to improve the levels of adiponectin could be very useful both to prevent the infection and to take care of the patients, improving their outcomes.

Given the similar pathway of action, it can be hypothesized that adiponectin and ω-3-PUFA could be used as real drugs to reduce inflammation, reducing both IL-6 and TNF-α levels as well as ameliorating the lung damage that occurs following coronavirus infection.

## Figures and Tables

**Table 1:** The principal antioxidants involved in lung infection and the immune-inflammatory response.

Dietary Supplementation	Main Natural Sources	Potential Effects in COVID-19 Infection
Flavonoids	Red wine, oranges, red fruits and vegetables	Reduces inflammation and immune response, blocking nuclear NF-κB translocation
Polyphenols	Green tea, broccoli, apples	
Vitamin C	Oranges, lemons, mangoes	
